# Case reports of two siblings with autism spectrum disorder and 15q13.3 deletions

**DOI:** 10.1002/npr2.12340

**Published:** 2023-06-01

**Authors:** Sawako Furukawa, Itaru Kushima, Branko Aleksic, Norio Ozaki

**Affiliations:** ^1^ Department of Psychiatry Nagoya University Graduate School of Medicine Nagoya Japan; ^2^ Medical Genomics Center Nagoya University Hospital Nagoya Japan; ^3^ Institute for Glyco‐core Research Nagoya University Nagoya Japan

**Keywords:** autism Spectrum disorder, chromosome 15q13.3 microdeletion syndrome, comparative genomic hybridization, DNA copy number variations, whole genome sequencing

## Abstract

**Background:**

Copy number variations (CNVs) have been implicated in psychiatric and neurodevelopmental disorders. Especially, 15q13.3 deletions are strongly associated with autism spectrum disorder (ASD), intellectual disability (ID), schizophrenia (SCZ), attention deficithyperactivity disorder (ADHD), and mood disorder.

**Case Presentation:**

We present two siblings with ASD. They had a father with bipolar disorder (BD). Patient 1 is a 21‐year‐old female with ASD and mild ID, who had language delay and repetitive behavior in childhood, social difficulties, and refused to go to school because of bullying. She was hospitalized in a psychiatric hospital several times. Patient 2 is a 19‐year‐old male with ASD and ADHD. He did not have developmental delay, but had social difficulties and impulsiveness, then refused to go to school because of bullying. He was treated by a psychiatrist for anxiety and disrupted sleep rhythms. Array comparative genomic hybridization was performed for the siblings and parents. 15q13.3 deletions were detected in the siblings and their healthy mothers. No other pathogenic CNVs were detected. We performed whole‐genome sequencing of the family and identified 13 rare missense variants in brain‐expressed genes, which may be responsible for the phenotypic differences between the siblings and their mother.

**Conclusions:**

This study shows incomplete penetrance and variable expressivity in 15q13.3 deletions. We detected second‐hit variants that may explain the phenotypic differences within this family. In addition, detecting 15q13.3 deletions may lead to early diagnosis and a better prognosis with careful follow‐up.

## Abstract 2

We present two siblings with autism spectrum disorder and 15q13.3 deletions. The 15q13.3 deletions were inherited from their healthy mother. This study shows incomplete penetrance and variable expressivity in 15q13.3 deletions.

## Graphical abstract

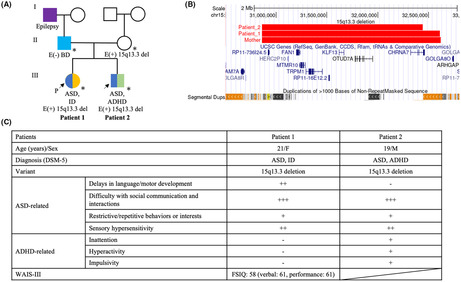

## 1 INTRODUCTION

Copy number variants (CNVs) have been implicated in psychiatric and neurodevelopmental disorders.^1^, ^2^, ^3^, ^4^, ^5^, ^6^, ^7^, ^8^ Specifically, 15q13.3 deletions have been identified as associated with intellectual disability (ID), autism spectrum disorder (ASD), schizophrenia (SCZ), attention deficit hyperactivity disorder (ADHD), and mood disorder.^9^, ^10^, ^11^, ^12^ 15q13.3 deletions are typically inherited (85.4%) and disproportionately of maternal origin.^12^ This region includes *CHRNA7*, which encodes the alpha‐7 subunit of the neuronal nicotinic acetylcholine receptor. Here, we present the first Japanese case report of two siblings (Patients 1 and 2) with ASD and 15q13.3 deletions. These CNVs were identified using array comparative genomic hybridization (aCGH) in our previous studies.^1^, ^2^

## 2 METHODS

### 2.1 Participants

All the members of this family were of Japanese ancestry. Patients were diagnosed according to the Diagnostic and Statistical Manual of Mental Disorders, Fifth Edition criteria for ASD, ID, and ADHD.

### 2.2 Genetic analysis

Genomic DNA was extracted from blood (Patient 1, Patient 2, and the mother) or saliva (the father) samples. aCGH was performed using NimbleGen 720 k Whole‐Genome Tiling Arrays (Roche NimbleGen). We generated CNV calls with Nexus Copy Number software, v9.0 (BioDiscovery).^1^ Previously, we confirmed that CNV calls from this array are highly accurate, with a validation rate of >99%. We also confirmed the CNV calls by whole‐genome sequencing (WGS) which explained next (Table S1).

To identify the genetic variants responsible for the phenotypic differences between the siblings and their mother, we performed WGS on this family using Complete Genomics technology. This sequencing was based on a nanoarray‐based short‐read sequencing‐by‐ligation technology.^13^ Ingenuity Variant Analysis bioinformatic software was used to detect the functional variants. Variants were kept only if they met the call confidence criteria of call quality ≥180, with read depth ≥ 10, and occurred outside the top 1% most exonic variable 100 base windows in healthy public genomes. Of these variants, we excluded those with a minor allele frequency ≥1% of the genomes in the Tohoku Medical Megabank Organization (ToMMo). We searched for paternal variants that the two siblings had in common. Finally, variants were only kept for the following analysis if they were nonsynonymous variants (i.e., missense variants, nonsense variants, frameshift variants, in‐frame indels). The pathogenicity of missense variants was assessed by PolyPhen‐2.^14^ All genomic locations are given in hg19 coordinates. We used the Human Brain Transcriptome database (https://hbatlas.org/pages/hbtd) to evaluate brain‐expressed genes.

### 2.3 Phenotypic analysis

We retrospectively collected clinical data of the two siblings with 15q13.3 deletions from their medical records. The data included developmental history, family history, medical history, psychiatric symptoms, history of hospitalizations, and medications. Based on the data, the severity of symptoms was graded as one of four levels by a board‐certified research psychiatrist: – (none), + (mildly present), ++ (moderately present), and +++ (strongly present).

## 3 CASE PRESENTATION

**FIGURE 1:**
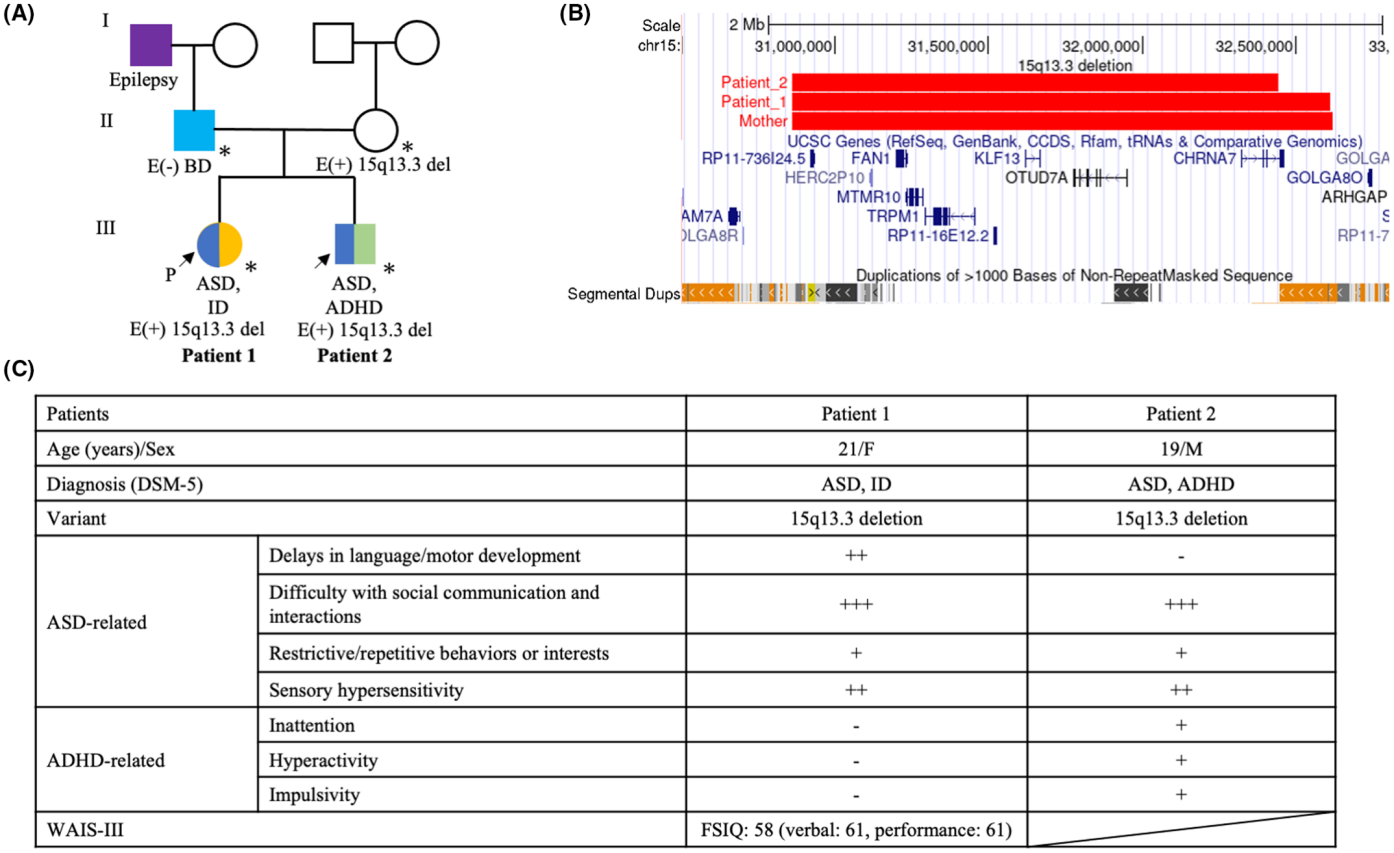
Clinical data of the patients and their parents. (A) The pedigree of this family. The 15q13.3 deletion was inherited from a healthy mother to two affected siblings (Patient 1 and patient 2). (B) Visualization of the 15q13.3 deletions in this family. The regions and sizes of the 15q13.3 deletions are, chr15:30861184–32 620 098, 1 758 914 bp (mother), chr15:30861184–32 610 833, 1 749 649 bp (Patient 1) and chr15:30861184–32 443 327, 1 582 143 bp (Patient 2). The genes affected by the 15q13.3 deletion are *RP11‐736I24.5*, *HERC2P10*, *FAN1*, *MTMR10*, *TRPM1*, *RP11‐16E12.2*, *KLF13*, *OTUD7A* and *CHRNA7*. (C) This table shows the summary of the clinical findings of the siblings. Although their CNV was the same, the phenotype of these patients was different at some points; only patient 1 had ID, and only patient 2 had ADHD. The severity of each psychiatric symptom was graded with 3 levels by a board‐certified research psychiatrist, based on the ADI‐R results: + (mildly present), ++ (moderately present), and +++ (strongly present). ADHD, attention deficit hyperactivity disorder; ADHD; DSM‐5, Diagnostic and Statistical Manual of Mental Disorders 5th Edition; ASD, autism spectrum disorder; BD, bipolar disorder; FSIQ, Full Scale IQ; ID, intellectual disability; WAIS‐III, Wechsler Adult Intelligence Scale‐III.

Figure 1 shows the pedigree of this family (1A), the 15q13.3 deletions identified (1B), and the clinical data of the two patients (1C). The patients had a family history of bipolar disorder (BD) (father) and epilepsy (paternal grandfather). The mother had no history of mental illness.

Patient 1 was a 21‐year‐old female with ASD and mild ID. She was born by elective cesarean delivery for breech presentation at term. Her birth weight was 3000 g. She did not have remarkable physical anomalies or diseases (e.g., epilepsy). She first walked at 16 months and said her first words at 2 years, language delay was noticed at a 3‐year checkup. In kindergarten, her movement was slower than other children, she was laid back and could not keep up with what the other preschoolers were doing. She was not good at getting along with her classmates and was often alone. In elementary school, her grades were poor and she experienced bullying. She could not keep up with the conversation of her classmates and often made unnecessary remarks, which sometimes left her out of the group. She had a keen interest in certain subjects, was often spinning around in circles in rooms, and was easily disturbed by the loud voice of classmates. In her early teens, she started to complain of dizziness and shortness of breath and refused to go to school. She visited a psychiatrist and was diagnosed with ASD. In high school, her relationship with her brother (Patient 2) worsened, and she became depressed and could not stand the sound of her family talking. She was treated in a psychiatric hospital with risperidone, sertraline, and alprazolam, with limited effect. She was admitted to the psychiatry department several times in her late teens to distance herself from the stressful home life. During her stay in the hospital, she was disturbed by the sounds of the televisions, the hair dryers, and the coughing of the other patients. Her IQ was 58 on the Wechsler Adult Intelligence Scale‐III (WAIS‐III). Her blood test and brain magnetic resonance imaging were not remarkable.

Patient 2 was the younger brother of Patient 1. He was a 19‐year‐old male with ASD and ADHD. He was also born by elective cesarean delivery at term. His birth weight was 2980 g. He did not have remarkable physical anomalies but had excessive myopia. He first walked and talked at 14 months. He had poor interactions with friends and was bullied. He was very sensitive even to small sounds and had a keen interest in cars. He was restless and hyperactive in elementary school. He frequently lost his belongings and crashed into the desk. He was clumsy. His grades in school were average. He experienced incontinence and enuresis throughout his childhood and suffered from stomachache and diarrhea since junior high. In junior high school, he refused to go to school because of bullying. He was treated by a psychiatrist for anxiety about interpersonal relationships and disrupted sleep rhythms. According to the clinical symptoms and developmental histories, he was diagnosed with ASD and ADHD. His blood test was not remarkable.

Both patients took Autism Diagnostic Interview – Revised (ADI‐R) to reinforce the ASD diagnosis. They met the criteria for autism in all four diagnostic domains, the domain A was significant; qualitative impairments in social interaction (Patient 1: 27, Patient 2: 22, cutoff: 10). During the clinical course, neither of the patients had psychotic symptoms.

We performed aCGH for the siblings and their parents; 15q13.3 deletions were detected in both siblings and their healthy mother. As neither of the two patients had other pathogenic CNVs, we hypothesized that rare (<1%) second‐hit variants (single nucleotide variants and insertions/deletions) may explain the phenotypic differences. We performed WGS of the siblings and their parents to identify potential second‐hit variants from rare variants inherited from their father with BD. Seventeen missense variants were detected in both affected siblings, all of which were predicted to be pathogenic by PolyPhen‐2 and inherited from their father. Among these variants, 13 were located in brain‐expressed genes (e.g., *KCND3*, *SEC24B*, and *TSPYL4*; Table S2). Maternally inherited variants and variants that the siblings did not share are shown in Tables S3 and S4.

## 4 DISCUSSION

15q13.3 deletions have incomplete penetrance in neuropsychiatric diagnosis (80.5%),^12^ including ASD (~35%).^15^ Consistent with this, 15q13.3 deletion in Patients 1 and 2 was inherited from their healthy mother (Figure 1A). In addition, the variable expressivity of 15q13.3 deletions is reported.^3^ This family showed variable expressivity; Patient 1 had ASD, ID and Patient 2 had ASD, ADHD (Figure 1A). To explain the incomplete penetrance and the variable expressivity, we assessed the second‐hit variants. We assumed that the pathogenic variants associated with the phenotypic differences were inherited from their father with BD because their mother with 15q13.3 deletion was healthy. We identified rare pathogenic missense variants in 13 brain‐expressed genes, which were shared between the two siblings and inherited from their father. Among these genes, *COL28A1* and *KCND3* were associated with ASD.^16^, ^17^, ^18^ These variants could cause phenotypic differences between siblings and their healthy mother. However, there are limitations to estimating second‐hit variants. First, the prediction of the pathogenicity of variants by in silico analysis is limited in terms of accuracy. In vitro functional analysis is required to determine the pathogenicity of these variants more accurately. Second, although we did not examine it, an aggregate effect of common variants (polygenic risk) may be involved in the phenotypic differences in this family.^19^

Finally, 15q13.3 deletions are strongly associated with the SCZ risk.^20^ Although our patients did not have psychotic symptoms, they may develop SCZ in the future. Detecting 15q13.3 deletions may lead to earlier diagnosis and a better prognosis of SCZ in young patients with careful follow‐up.

## AUTHOR CONTRIBUTIONS

S.F. and I.K. designed the study. I.K. and B.A. performed genetic analysis. S.F., I.K., and N.O. recruited the participants and/or collected DNA samples or phenotype data. S.F. and I.K. wrote the first draft of the manuscript, and the others commented on and refined it. All the authors carefully read the manuscript and approved the final version.

## ETHICS APPROVAL

This study was approved by the ethics committee of Nagoya University Graduate School of Medicine; Genomic study for brain and mental disorders and effectiveness and side effect of pharmacological treatment (2010–1033). This study complied with all the provisions of the Declaration of Helsinki.

Informed consent: Written informed consent was obtained from all the participants.

Registry and the Registration No. of the study/trial: N/A

Animal Studies: N/A

## FUNDING INFORMATION

This research was supported by research grants from the Ministry of Education, Culture, Sports, Science and Technology of Japan (MEXT) and the Ministry of Health, Labour and Welfare of Japan; the Japan Agency for Medical Research and Development (AMED) under Grant Nos. JP20dm0107087, JP21wm0425007, JP21dm0207075, JP21ak0101113, JP21dk0307075, JP20dk0307081, JP21dk0307103, JP21ek0109488, JP21km0405216, JP21ek0109411, and JP22tm0424222; the Japan Society for the Promotion of Science (JSPS) KAKENHI Grant Nos. 17H05090, 18H04040, 21 K07543, 21H00194, 21H04815, 18 K07590, and 15 K19720; and the SENSHIN Medical Research Foundation.

## CONFLICT OF INTEREST STATEMENT

S.F. and I.K. declare no conflicts of interest. N.O. has received research support or speakers' honoraria from, or has served as a consultant to, Sumitomo Dainippon, Eisai, Otsuka, KAITEKI, Mitsubishi Tanabe, Shionogi, Eli Lilly, Mochida, DAIICHI SANKYO, Nihon Medi‐Physics, Takeda, Meiji Seika Pharma, EA Pharma, Pfizer, MSD, Lundbeck Japan, Tsumura, Novartis, Boehringer Ingelheim, Viatris, Kyowa, Janssen, Yoshitomi Yakuhin, Kyowa Kirin, Ono, Astellas, UCB, Taisho Toyama, Medical Review, and Woolsey, outside the submitted work.

## Supporting information


Data S1.

## Data Availability

The data that support the findings of this study are available in this article and its supporting information files.
