# Supplementary material for: Case reports of two siblings with autism spectrum disorder and 15q13.3 deletions
Source: Neuropsychopharmacol Rep. 2023 Jun 1;43(3):462–6. doi: 10.1002/npr2.12340 (PMC10496043; doi:10.1002/npr2.12340)
Supplement: Supplementary file 1 — Data S1. [file NPR2-43-462-s001.docx]

**Supporting Information**

**Detecting brain-expressed genes**

We used Human Brain Transcriptome database (https://hbatlas.org/pages/hbtd) to evaluate brain-expressed genes. We used a basic gene search for “Brain regions”. After getting the graphic outputs, if any graph exceeded Signal intensity (Log2) = 5, we considered the gene to be actively expressed in the brain.

**Maternally inherited variants**

We also searched for potential second hit variants from rare variants inherited from their mother. We identified rare pathogenic missense variants in 12 brain-expressed genes, which were shared between the two siblings and inherited from their mother (Table S3). Among these genes, *SBF1* was associated with ASD (1, 2). However, the mother who had the variant in this gene did not have ASD.

***De novo* variants**

We did not find possible *de novo* variants in both Patient 1 and Patient 2 in the coding region.

**Variants that the siblings did not share**

We also checked for variants which the siblings did not share. We found 2 loss of function variants that only Patient 2 had (Table S4).

**Table S1. The CNVs confirmed by whole genome sequencing**

Table S1a. Patient 1

| **chr** | **begin** | **end** | **Ploidy** | **CNVType** | **Gene** |
| --- | --- | --- | --- | --- | --- |
| chr15 | 30596000 | 30602000 | 2 | = |  |
| chr15 | 30602000 | 30916000 | N | hypervariable |  |
| chr15 | 30916000 | 30944000 | 1 | - | *ARHGAP11B;LOC100132726* |
| chr15 | 30944000 | 30950000 | N | hypervariable |  |
| chr15 | 30950000 | 30978000 | 1 | - | *LOC100288637* |
| chr15 | 30978000 | 30988000 | N | hypervariable |  |
| chr15 | 30988000 | 31102000 | 1 | - | *LOC100288637;LOC100507035;LOC390561* |
| chr15 | 31102000 | 31104000 | N | hypervariable |  |
| chr15 | 31104000 | 31106000 | N | invariant |  |
| chr15 | 31106000 | 31112000 | N | hypervariable |  |
| chr15 | 31112000 | 31136000 | 1 | - | *LOC390561* |
| chr15 | 31136000 | 31140000 | N | hypervariable |  |
| chr15 | 31140000 | 31148000 | N | invariant |  |
| chr15 | 31148000 | 31910000 | 1 | - | *KLF13;LOC100506669;LOC283710;MIR211;MTMR10;MTMR15;OTUD7A;TRPM1* |
| chr15 | 31910000 | 31924000 | N | hypervariable |  |
| chr15 | 31924000 | 31984000 | 1 | - | *OTUD7A* |
| chr15 | 31984000 | 31990000 | N | hypervariable |  |
| chr15 | 31990000 | 31996000 | 1 | - |  |
| chr15 | 31996000 | 32016000 | N | hypervariable |  |
| chr15 | 32016000 | 32446000 | 1 | - | *CHRNA7* |
| chr15 | 32446000 | 32510000 | N | hypervariable |  |
| chr15 | 32510000 | 32518000 | 1 | - |  |
| chr15 | 32518000 | 32548000 | N | hypervariable |  |
| chr15 | 32548000 | 32556000 | 2 | = |  |
| chr15 | 32556000 | 32898000 | N | hypervariable |  |
| chr15 | 32898000 | 32904000 | 2 | = |  |

Table S1b. Patient 2

| **chr** | **begin** | **end** | **Ploidy** | **CNVType** | **Gene** |
| --- | --- | --- | --- | --- | --- |
| chr15 | 30596000 | 30602000 | 2 | = |  |
| chr15 | 30602000 | 30916000 | N | hypervariable |  |
| chr15 | 30916000 | 30944000 | 1 | - | *ARHGAP11B;LOC100132726* |
| chr15 | 30944000 | 30950000 | N | hypervariable |  |
| chr15 | 30950000 | 30978000 | 1 | - | *LOC100288637* |
| chr15 | 30978000 | 30988000 | N | hypervariable |  |
| chr15 | 30988000 | 31102000 | 1 | - | *LOC100288637;LOC100507035;LOC390561* |
| chr15 | 31102000 | 31112000 | N | hypervariable |  |
| chr15 | 31112000 | 31136000 | 1 | - | *LOC390561* |
| chr15 | 31136000 | 31140000 | N | hypervariable |  |
| chr15 | 31140000 | 31148000 | N | invariant |  |
| chr15 | 31148000 | 31910000 | 1 | - | *KLF13;LOC100506669;LOC283710;MIR211;MTMR10;MTMR15;OTUD7A;TRPM1* |
| chr15 | 31910000 | 31924000 | N | hypervariable |  |
| chr15 | 31924000 | 31984000 | 1 | - | *OTUD7A* |
| chr15 | 31984000 | 31990000 | N | hypervariable |  |
| chr15 | 31990000 | 31996000 | 1 | - |  |
| chr15 | 31996000 | 32016000 | N | hypervariable |  |
| chr15 | 32016000 | 32446000 | 1 | - | *CHRNA7* |
| chr15 | 32446000 | 32510000 | N | hypervariable |  |
| chr15 | 32510000 | 32518000 | 1 | - |  |
| chr15 | 32518000 | 32548000 | N | hypervariable |  |
| chr15 | 32548000 | 32556000 | 2 | = |  |
| chr15 | 32556000 | 32898000 | N | hypervariable |  |
| chr15 | 32898000 | 32904000 | 2 | = |  |

Table S1c. Mother

| **chr** | **begin** | **end** | **Ploidy** | **CNVType** | **Gene** |
| --- | --- | --- | --- | --- | --- |
| chr15 | 30596000 | 30602000 | 2 | = |  |
| chr15 | 30602000 | 30916000 | N | hypervariable |  |
| chr15 | 30916000 | 30944000 | 1 | - | *ARHGAP11B;LOC100132726* |
| chr15 | 30944000 | 30950000 | N | hypervariable |  |
| chr15 | 30950000 | 30978000 | 1 | - | *LOC100288637* |
| chr15 | 30978000 | 30988000 | N | hypervariable |  |
| chr15 | 30988000 | 31102000 | 1 | - | *LOC100288637;LOC100507035;LOC390561* |
| chr15 | 31102000 | 31104000 | N | hypervariable |  |
| chr15 | 31104000 | 31106000 | N | invariant |  |
| chr15 | 31106000 | 31112000 | N | hypervariable |  |
| chr15 | 31112000 | 31136000 | 1 | - | *LOC390561* |
| chr15 | 31136000 | 31140000 | N | hypervariable |  |
| chr15 | 31140000 | 31148000 | N | invariant |  |
| chr15 | 31148000 | 31910000 | 1 | - | *KLF13;LOC100506669;LOC283710;MIR211;MTMR10;MTMR15;OTUD7A;TRPM1* |
| chr15 | 31910000 | 31924000 | N | hypervariable |  |
| chr15 | 31924000 | 31984000 | 1 | - | *OTUD7A* |
| chr15 | 31984000 | 31990000 | N | hypervariable |  |
| chr15 | 31990000 | 31996000 | 1 | - |  |
| chr15 | 31996000 | 32016000 | N | hypervariable |  |
| chr15 | 32016000 | 32446000 | 1 | - | *CHRNA7* |
| chr15 | 32446000 | 32510000 | N | hypervariable |  |
| chr15 | 32510000 | 32518000 | 1 | - |  |
| chr15 | 32518000 | 32548000 | N | hypervariable |  |
| chr15 | 32548000 | 32556000 | 1 | - |  |
| chr15 | 32556000 | 32898000 | N | hypervariable |  |
| chr15 | 32898000 | 32904000 | 2 | = |  |

Table S1d. Father

| **chr** | **begin** | **end** | **Ploidy** | **CNVType** | **Gene** |
| --- | --- | --- | --- | --- | --- |
| chr15 | 30596000 | 30602000 | 2 | = |  |
| chr15 | 30602000 | 30916000 | N | hypervariable |  |
| chr15 | 30916000 | 30944000 | 2 | = |  |
| chr15 | 30944000 | 30950000 | N | hypervariable |  |
| chr15 | 30950000 | 30978000 | 2 | = |  |
| chr15 | 30978000 | 30988000 | N | hypervariable |  |
| chr15 | 30988000 | 31102000 | 2 | = |  |
| chr15 | 31102000 | 31112000 | N | hypervariable |  |
| chr15 | 31112000 | 31136000 | 2 | = |  |
| chr15 | 31136000 | 31146000 | N | hypervariable |  |
| chr15 | 31146000 | 31910000 | 2 | = |  |
| chr15 | 31910000 | 31924000 | N | hypervariable |  |
| chr15 | 31924000 | 31984000 | 2 | = |  |
| chr15 | 31984000 | 31990000 | N | hypervariable |  |
| chr15 | 31990000 | 31996000 | 2 | = |  |
| chr15 | 31996000 | 32016000 | N | hypervariable |  |
| chr15 | 32016000 | 32446000 | 2 | = |  |
| chr15 | 32446000 | 32510000 | N | hypervariable |  |
| chr15 | 32510000 | 32518000 | 2 | = |  |
| chr15 | 32518000 | 32548000 | N | hypervariable |  |
| chr15 | 32548000 | 32556000 | 2 | = |  |
| chr15 | 32556000 | 32898000 | N | hypervariable |  |
| chr15 | 32898000 | 32904000 | 2 | = |  |

**Table S2. 13 rare missense paternal variants in brain-expressed genes identified in the study**

Variants were shared by the siblings.

| **Position (hg19)** | **Gene Symbol** | **Variants** | **Genotype** | **PolyPhen-2 Function Prediction** | **dbSNP ID** | **Japanese Population** |
| --- | --- | --- | --- | --- | --- | --- |
| 7:7480479 | *COL28A1* | NM_001037763.3:c.1664A>C:p.K555T | Het | Probably Damaging | 150698780 | 0.006 |
| 1:112525257 | *KCND3* | NM_004980.5:c.92C>T:p.P31L | Het | Probably Damaging | 374337721 |  |
| 4:110384381 | *SEC24B* | NM_006323.5:c.458C>T:p.S153F | Het | Possibly Damaging | 746946726 | 0.005 |
| 6:31733709 | *VWA7* | NM_025258.3:c.2450C>T:p.P817L | Het | Possibly Damaging | 759300576 | 0.0002 |
| 6:116574483 | *TSPYL4* | NM_021648.5:c.689G>C:p.R230P | Het | Probably Damaging | 1424718594 |  |
| 11:64678099 | *ATG2A* | NM_015104.3:c.1696C>T:p.R566C | Het | Possibly Damaging | 140600460 |  |
| 11:85407355 | *SYTL2* | NM_206930.3:c.823A>T:p.N820Y | Het | Possibly Damaging | 1240521316 | 2.00E-04 |
| 19:46124782 | *EML2* | NM_001193268.3:c.1558G>T:p.V319F | Het | Possibly Damaging | 184799000 | 0.006 |
| 19:51323274 | *KLK1* | NM_002257.4:c.514C>T:p.L172F | Het | Possibly Damaging | 193199844 | 2.00E-03 |
| 20:61597920 | *SLC17A9* | NM_022082.4:c.1105G>A:p.G369S | Het | Probably Damaging | 186348088 | 0.001 |
| 22:24567727 | *CABIN1* | NM_001199281.1:c.5804G>A:p.R1885Q | Het | Probably Damaging | 376189661 |  |
| 22:50659874 | *TUBGCP6* | NM_020461.4:c.2914G>C:p.A972P | Het | Possibly Damaging | 199978914 | 2.00E-03 |
| 22:50921144 | *ADM2* | NM_001253845.2:c.259C>T:p.R87C | Het | Possibly Damaging | 775358101 |  |

**Table S3. 12 rare missense maternal variants in brain-expressed genes identified in the study**

| **Position (hg19)** | **Gene Symbol** | **Variants** | **Genotype** | **PolyPhen-2 Function Prediction** | **dbSNP ID** | **Japanese Population** |
| --- | --- | --- | --- | --- | --- | --- |
| 13:75861080 | *TBC1D4* | NM_014832.3:c.3556C>T:p.R1186W | Het | Possibly Damaging | 201344804 | 0.003 |
| 8:142229914 | *SLC45A4* | NM_001286648.1:c.445G>A:p.D149N | Het | Probably Damaging | 766115317 | 0.0002 |
| 1:35921763 | *KIAA0319L* | NM_024874.4:c.1507C>G:p.P503A | Het | Probably Damaging | 778787484 |  |
| 2:24245720 | *MFSD2B* | NM_001080473.1:c.1007G>C:p.W336S | Het | Probably Damaging | 188718007 | 0.006 |
| 2:119726796 | *MARCO* | NM_006770.3:c.158T>C:p.L53P | Het | Probably Damaging | 1208848705 | 0.0008 |
| 10:116197636 | *ABLIM1* | NM_001003407.1:c.2010A>C:p.K670N | Het | Probably Damaging | 1190121662 | 0.0002 |
| 16:2053617 | *ZNF598* | NM_178167.3:c.335G>A:p.R112K | Het | Possibly Damaging | 865915732 | 0.0001 |
| 16:30594109 | *ZNF785* | NM_152458.6:c.990C>A:p.H330Q | Het | Probably Damaging | 141592630 | 0.007 |
| 18:65181182 | *DSEL* | NM_032160.2:c.694C>T:p.H232Y | Het | Probably Damaging |  | 0.0005265 |
| 21:45547837 | *PWP2* | NM_005049.2:c.2165C>T:p.T722M | Het | Probably Damaging | 144002518 | 0.0001 |
| 22:50885659 | *SBF1* | NM_002972.2:c.5594C>T:p.T1865M | Het | Probably Damaging | 375012426 | 0.007 |
| 13:28494372 | *PDX1* | NM_000209.3:c.97C>G:p.P33A | Het | Probably Damaging | 192902098 | 0.003 |

Variants were shared by the siblings.

**Table S4. Rare pathogenic nonsense variants in brain-expressed genes which the siblings did not share**

| **Position (hg19)** | **Gene Symbol** | **Variants** | **Genotype** | **Inheritance** | **Patient** | **Translation Impact** | **Japanese Population** |
| --- | --- | --- | --- | --- | --- | --- | --- |
| 8:95952165 | *TP53INP1* | NM_001135733.1:c.394T>A:p.Y132* | Het | maternal | 2 | stop gain |  |
| 16:66600713 | *CMTM1;  CKLF-CMTM1* | NM_052999.4:c.282_283insA:p.T100fs*2 | Het | paternal | 2 | frameshift |  |

**References**

1. O'Roak BJ, Vives L, Girirajan S, Karakoc E, Krumm N, Coe BP, et al. Sporadic autism exomes reveal a highly interconnected protein network of de novo mutations. Nature. 2012;485(7397):246-50.

2. Zhou X, Feliciano P, Shu C, Wang T, Astrovskaya I, Hall JB, et al. Integrating de novo and inherited variants in 42,607 autism cases identifies mutations in new moderate-risk genes. Nat Genet. 2022;54(9):1305-19.
